# Microorganism changes in the gut of *Apis mellifera* surviving for the long term in *Camellia oleifera* forests

**DOI:** 10.3389/fcimb.2025.1608835

**Published:** 2025-06-12

**Authors:** Longsheng Chen, Zhen Li, Dongju Yuan, Yongzhong Chen, Yanming Xu, Wei Tang, Caixia Liu

**Affiliations:** ^1^ Hunan Academy of Forestry, National Engineering Research Center of Oil-Tea Camellia, Yuelushan Laboratory, Changsha, Hunan, China; ^2^ College of Life Science and Resources and Environment, Yichun University, Yichun, Jiangxi, China; ^3^ Hunan Linkeda Agricultural and Forestry Technology Service Co., Ltd., Changsha, Hunan, China

**Keywords:** honey bee, *Gilliamella apicola*, GH4, pollinate, microbial structure

## Abstract

Alpha-galactosides (oligosaccharides) in *C. oleifera* nectar and pollen cause honey bee larval rot and worker bloats. Honey bee colonies surviving in *C. oleifera* forests for a long period have low rates of larval rot and worker bloats; however, the mechanism of oligosaccharide metabolism is unclear. In this study, we used metagenomics and metabolomics to investigate the structure and function of the gut flora and the digestion characteristics of oligosaccharides in the gut of *A. mellifera* foragers (CN group) that had been in the *C. oleifera* forest for a long period (continuously for 14 years), and those that had not been pollinated with *C. oleifera* (N group) after 24 h of consumption of *C. oleifera* honey. The results revealed that the abundance of *Gilliamella apicola* up to 24.08%, which can metabolize α-galactoside (α-Gal), was significantly higher (*P* < 0.05) in the gut of foragers in the CN group than in the N group. Additionally, the gut flora of foragers in the CN group carried a significantly higher (*P* < 0.05) abundance of genes encoding α-galactosidase (Glycoside hydrolase family 4, GH4) than the N group. Similarly, metabolomic results indicated that the three toxic oligosaccharides in *C. oleifera* honey were lower in the gut of CN group foragers. These results suggest that the gut flora of *A. mellifera*, which inhabits oil tea forests for long periods of time, changes and adapts to the predominant ecological niche, enhancing the host’s ability to metabolize toxic oligosaccharides. This important discovery provides positive guidance for the subsequent directions for breeding of *A. mellifera* (*G. apicola* enrichment and GH4 upregulation), specialized in pollinating *C. oleifera*.

## Introduction

*Camellia oleifera* is an important woody edible oil tree species native to China that is now widely distributed in East and Southeast Asian countries, with a cultivation history of up to 2,300 years (Luan et al., 2020). The fatty oil of *C. oleifera* seeds is called camellia oil, and its unsaturated fatty acid content is up to 90% or more, with linoleic acid content as high as 75%–83% and linoleic acid content reaching 7.4%–13% (Gao et al., 2018). Camellia oil contains unsaturated fatty acids that reduce the risk of cardiovascular disease, boost immunity, lower cholesterol, prevent and treat high blood pressure, and prevent cancer (Li et al., 2020). Food and Agriculture Organization of the United Nations has promoted camellia oil as a high-grade healthcare edible oil due to its similar chemical composition to olive oil (Liang et al., 2017).

*C. oleifera* breeding is characterized by a typical late-acting self-incompatible. After self-pollination, pollen tubes can cross the style to reach the ovary; however, they still experience self-fertilization failure (Li et al., 2023). Therefore, *C. oleifera* is a heterogamous pollinator that must use a pollinator to bear fruit. According to statistics, more than 130 kinds of pollinating insects for *C. oleifera* are present in traditionally planted *C. oleifera* forests, with *Andrena camellia* and *Colletes gigas* being the dominant pollinating insects (Hu et al., 2019). However, cultivation has recently adopted the “deforestation planting” method due to single-crop cultivation in the *C. oleifera* forest. The misuse of herbicides has seriously damaged the habitat of wild pollinators. As a result, the species and the number of pollinating insects in *C. oleifera* forests have sharply reduced, leading to a further decline in fruit set rates (Li et al., 2024). After applying commercially reared honey bees with multiple hive spleens (*Apis cerana* and *Apis mellifera*) to pollinate *C. oleifera*, poisoning symptoms, including honey bee larvae rotting and adult bee bloating, are observed. Consequently, honey bees are reluctant to collect nectar and pollen from *C. oleifera* flowers (Li et al., 2022). Recent studies have demonstrated that high concentrations of manninotriose, raffinose, and stachyose in *C. oleifera* honey are responsible for the poisoning of larvae and adult worker bees by *A. mellifera* (Li et al., 2022).

Symbiotic bacteria in the gut of the honey bee help the host itself metabolize toxic sugars and complex carbohydrates (Zheng et al., 2016, 2019). The rotting rate of *A. mellifera* honey bee colonies is 0.12% after long-term survival in *C. oleifera* forests for more than 14 years, and worker bees do not exhibit symptoms of bloating (He et al., 2019). Accordingly, the gut flora of *A. mellifera* may play an important metabolic role in this adaptation. In this study, metagenome and metabolomics were employed to characterize the changes in the gut flora of *A. mellifera* worker bees in response to manninotriose, raffinose, and stachyose in *C. oleifera* honey. This study aimed to reveal the molecular mechanisms underlying the adaptation of *A. mellifera* workers to toxic oligosaccharides in *C. oleifera* honey in long-term *C. oleifera* forests. The findings of this study are significant for the cultivation of *C. oleifera* pollinating honey bees and provide novel insights to improve *C. oleifera* pollination efficiency.

## Materials and methods

### Insect rearing

Three *A. mellifera* colonies with swarming potential to survive in the *C. oleifera* plantation for 14 years were selected as treatment groups in Shengqiao Town, Changning City, Hunan Province, China (112.40 °N, 26.43 °E). The *C. oleifera* trees are over twenty years old and growing well. Thirty foragers were collected from each colony and kept in three sterile cup cages with ventilation holes. All honey bees were subjected to a 2 h fasting period prior to feeding. Then, adequate *C. oleifera* honey (42.5°Bé) was added to each cup and fed for 24 h. These cup cages containing worker bees were kept in an incubator at 34.5°C with 75% relative humidity. The honey bees in this group are referred to as the CN group.

The control group comprised the same three swarming potentials from *A. mellifera* colonies raised in Tianxin District, Changsha City, Hunan Province, China (116.25 °N, 40.01 °E). However, these colonies did not pollinate *C. oleifera* before. Sixty foragers were collected from each colony and distributed equally between two sterile cup cages, constituting 180 bees in six cup cages. One group comprised three cup cages from three different colonies in six cup cages. Therefore, they were divided into two groups. All honey bees were starved for 2 hbefore feeding. One group was fed adequate *C. oleifera* honey (concentration, 42.5°Bé), referred to as group N, and the other group was fed adequate 50% sterile sucrose solution, referred to as group CK. Both groups were fed for 24 h. These cup cages containing worker bees were kept in an incubator at 34.5°C with 75% relative humidity.

### Collection of gut tissue samples from workers

After 24 h of feeding, the intact guts of honey bees from each of the three groups were dissected, and the honey sacs were removed using sterile forceps and placed in a 1.5 mL sterile centrifuge tube. Each sample consisted of six honey bee guts from three cup cages, with two honey bees originating from the same cup cage. Three biological replicates were used for each group. Two samples were dissected two times to obtain two samples: one for metagenomic sequencing and the other for metabolomic analysis. All samples were collected, frozen in liquid nitrogen for 1 h, and transferred to a –80°C refrigerator for subsequent detection.

### Chemicals and reagents

Methanol, acetonitrile, ethanoic acid, and water were obtained from Fisher Scientific, Inc. (Waltham, MA, United States).

### Gut flora structural analysis and functional annotation

DNA was extracted from the gut bacterial using an E.Z.N.A. Soil DNA Kit (Omega Bio-tek, Inc., United States) following the manufacturer’s instructions. Sequencing and data analysis were performed by Beijing Allwegene Technology Co., Ltd. Metagenome data quality control, assembly and annotation of foragers gut flora can be found in the Supporting Information.

### Alpha-diversity analysis of gut bacteria

Shannon index, a measurement index based on information theory, is widely used in ecology. The formula is H=-ΣPilnPi, where H represents the diversity index and Pi represents the relative abundance of the ith species. Shannon index is used to measure the diversity of the community. The larger the Shannon index, the higher the diversity of the species; conversely, the smaller the Shannon index, the lower the diversity of the species.

Simpson index is calculated as C=ΣP_i_, where C is the concentration of species (the maximum value is 1), and P_i_ is the ratio of the number of individuals of the ith taxon in the whole. In general, the Simpson index has a range of values between 0 and 1, with larger values indicating greater diversity and smaller values indicating less diversity.

### Wide-targeted analysis of metabolites

#### Pre-treatment of gut samples

The intact guts sample was removed from the –80°C freezer and thawed slowly at 4°C. An appropriate amount of the sample was added to a pre-cooled MeOH: ACN: H_2_O solution containing an internal standard (v: v: v = 2:2:1) and two steel balls. Gut samples were preprocessed with reference to the method of (Xu et al., 2024). The supernatant was aspirated into an injection bottle for liquid chromatography-mass spectrometry (LC-MS-MS) analysis. Additionally, 10 μL of each sample was mixed to prepare a QC sample packed into an injection bottle.

### LC-MS/MS analysis

All metabolite separations were performed using an ultra-performance liquid chromatography (UPLC) system (SCIEX, UK) coupled to Orbitrap Exploris 120 mass spectrometer (Orbitrap, Thermo Fisher Scientific) by Beijing Allwegene Technology Co., Ltd. Specific chromatography, mass spectrometry detection conditions, metabolites data annotation and analysis were as described in the Supporting Information.

### Statistical methods

All statistical analyses were performed using SPSS software version 23 (IBM Corp, Armonk, NY). Results are expressed as mean ± standard error of the mean (SEM). Differences between groups were tested for statistical significance using Student’s t-test or analysis of variance (ANOVA) and Fisher’s least squares difference (PLSD) test. Significance levels are shown and *P* < 0.05 is considered to be a significant difference between the two groups.

## Results

### Data quality and gut flora alpha-diversity in CK, N, and CN

**Figure 1:**
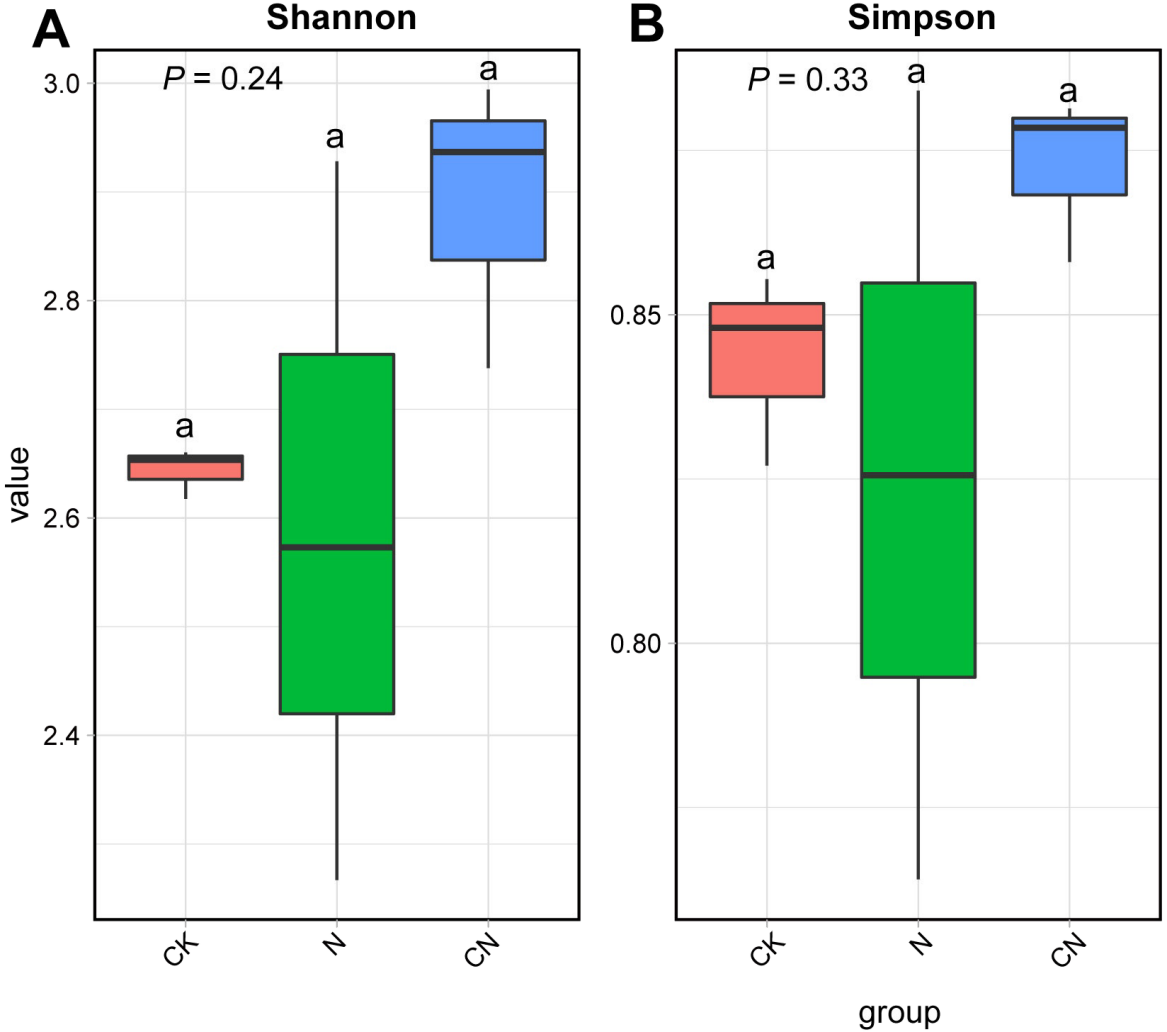
Alpha diversity of gut flora in control *A. mellifera* (CK), normal *A. mellifera* (N) and Changning *A. mellifera* (CN). **(A)** Shannon index; **(B)** Simpson index. The same lowercase letter in the three columns indicates data no significant difference (*P* > 0.05), and different lowercase letters indicate data significant differences (*P* < 0.05).

The average number of clean reads for the nine samples was 43,911,068, with an average clean base of 6.12 G. Q20 (represents a quality value of 20 if a base is 99% correct) had an average of 97.89%, and Q30 (represents a quality value of 20 if a base is 99% correct) had an average of 93.46%, indicating high sequencing quality (**Table 1**). **Figure 1** reveals that the differences in the Shannon and Simpson among the three groups were not significant (F_2, 6_ = 1.80, df = 2, *P* = 0.24; F_2, 6_ = 1.32, df = 2, *P* = 0.33; F_2, 6_ = 1.31, df = 2, *P* = 0.34), indicating that the changes in the diversity of flora in the gut of *Apis mellifera* foragers were insignificant in CK, N, and CN groups.

**Table 1:** Statistics quality of metagenome sequencing.

Samples	Clean reads	Clean bases (GB)	GC Content	Q20	Q30
CK_1	44 368 952	6.1875	40.84%	97.85%	93.38%
CK_2	41 850 642	5.8326	40.75%	98.11%	94.04%
CK_3	44 100 472	6.1467	42.56%	97.95%	93.68%
N_1	44 991 020	6.2749	44.09%	98.00%	93.83%
N_2	42 968 454	5.9933	42.50%	97.91%	93.50%
N_3	43 238 504	6.0299	41.54%	97.67%	92.99%
CN_1	43 483 168	6.0627	37.34%	97.87%	93.36%
CN_2	43 852 450	6.1158	36.99%	97.70%	92.97%
CN_3	46 345 950	6.4628	38.20%	97.92%	93.47%

### Gut flora structure in CK, N, and CN

**Figure 2:**
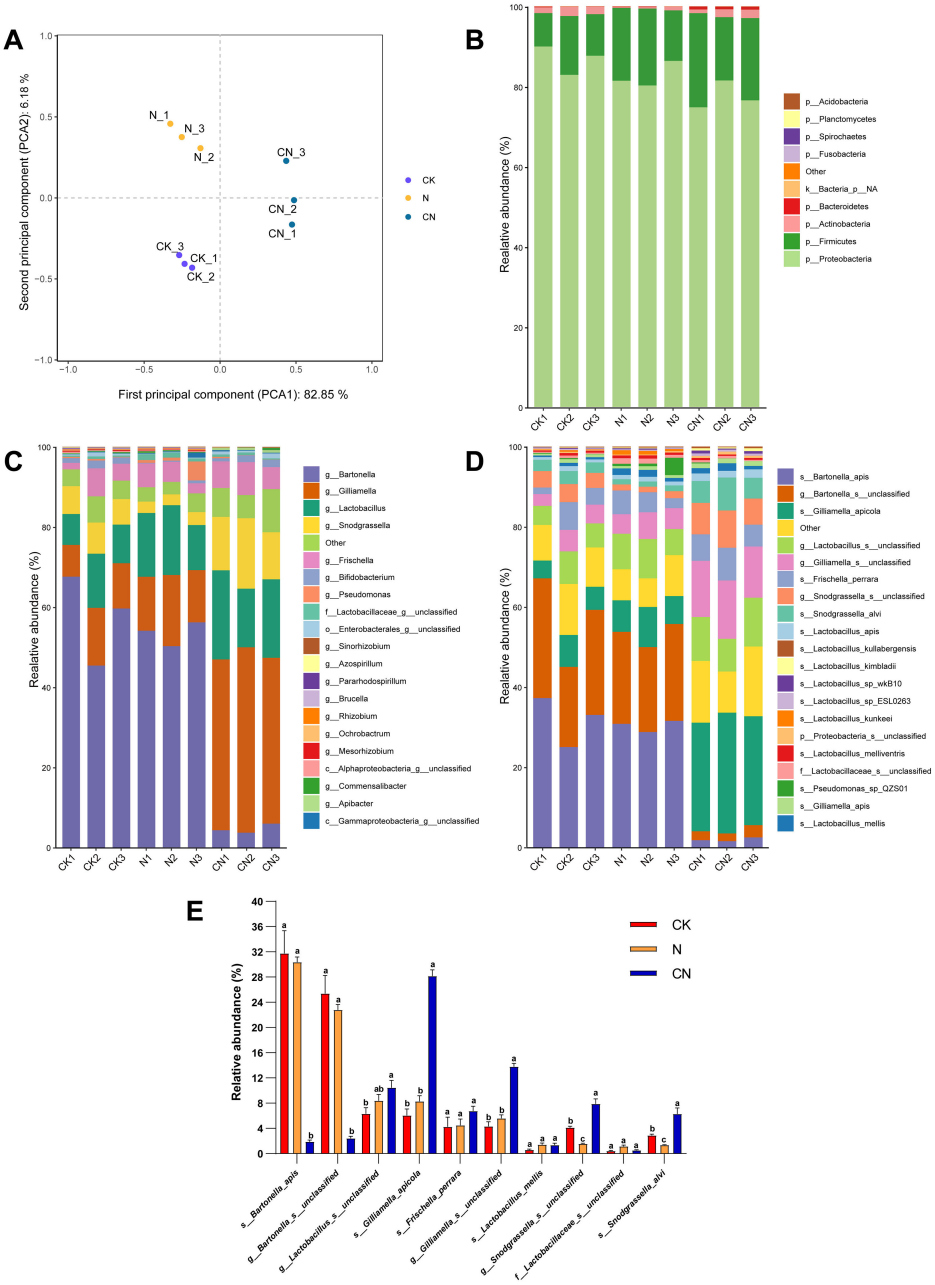
Gut flora composition analysis of *A. mellifera* from three groups. **(A)** PCA analysis results; **(B)** phylum level; **(C)** genus level; **(D)** species level; **(E)** top 10 dominant species. The same lowercase letter in the three columns of the same species indicates data no significant difference (*P* > 0.05), and different lowercase letters indicate data significant differences (*P* < 0.05).

PCA revealed significant changes in the structure of the intestinal flora of honey bees in CK, N, and CN groups (**Figure 2A**). Subsequent community bar graphs demonstrated that the top four dominant phyla in CK, N, and CN groups were Proteobacteria (CK, 86.84%; N, 82.67%; and CN, 77.60%), Firmicutes (CK, 11.12%; N, 16.67%; and CN, 19.93%), Actinobacteria (CK, 1.85%; N, 0.47%; and CN, 1.67%), and Bacteroidetes (CK, 0.12%; N, 0.06%; CN, and 0.07%). However, the dominant phyla varied considerably among the three groups (**Figure 2B**).

The top four dominant genus in the CK group were *Bartonella* (57.52%), *Gilliamella* (11.22%), *Lactobacillus* (10.28%), and *Snodgrassella* (7.04%) (**Figure 2C**). The top four dominant genera in the N group were *Bartonella* (53.46%), *Lactobacillus* (14.87%), *Gilliamella* (14.74%), and *Frischella* (4.49%). The top four dominant genera in the CN group were *Gilliamella* (43.43%), *Lactobacillus* (18.82%), *Snodgrassella* (14.21%), and *Frischella* (6.79%).

The top four dominant species in the CK group were *Bartonella apis* (31.76%), *g_Bartonella_s_unclassified* (25.38%), *g_Lactobacillus_s_unclassified* (6.30%), and *Gilliamella apicola* (6.06%) (**Figure 2D**). The top four dominant species in the N group were *Bartonella apis* (30.34%), *g_Bartonella_s_unclassified* (22.78%), *g_Lactobacillus_s_unclassified* (8.39%), and *Gilliamella apicola* (8.26%). The top four dominant species in the CN group were *Gilliamella apicola* (28.15%), *g_Gilliamella_s_unclassified* (13.79%), *g_Lactobacillus_s_unclassified* (10.44%), and *g_Snodgrassella_s_unclassified* (7.89%). **Figure 2E** illustrates the top 10 dominant species in each group, with *Gilliamella apicola* abundance being significantly higher (F_2, 6_ = 153.97, df = 2, *P* < 0.05) in the CN group than in CK and N groups.

### Carbohydrate annotation results for gut flora in CK, N, and CN

**Figure 3:**
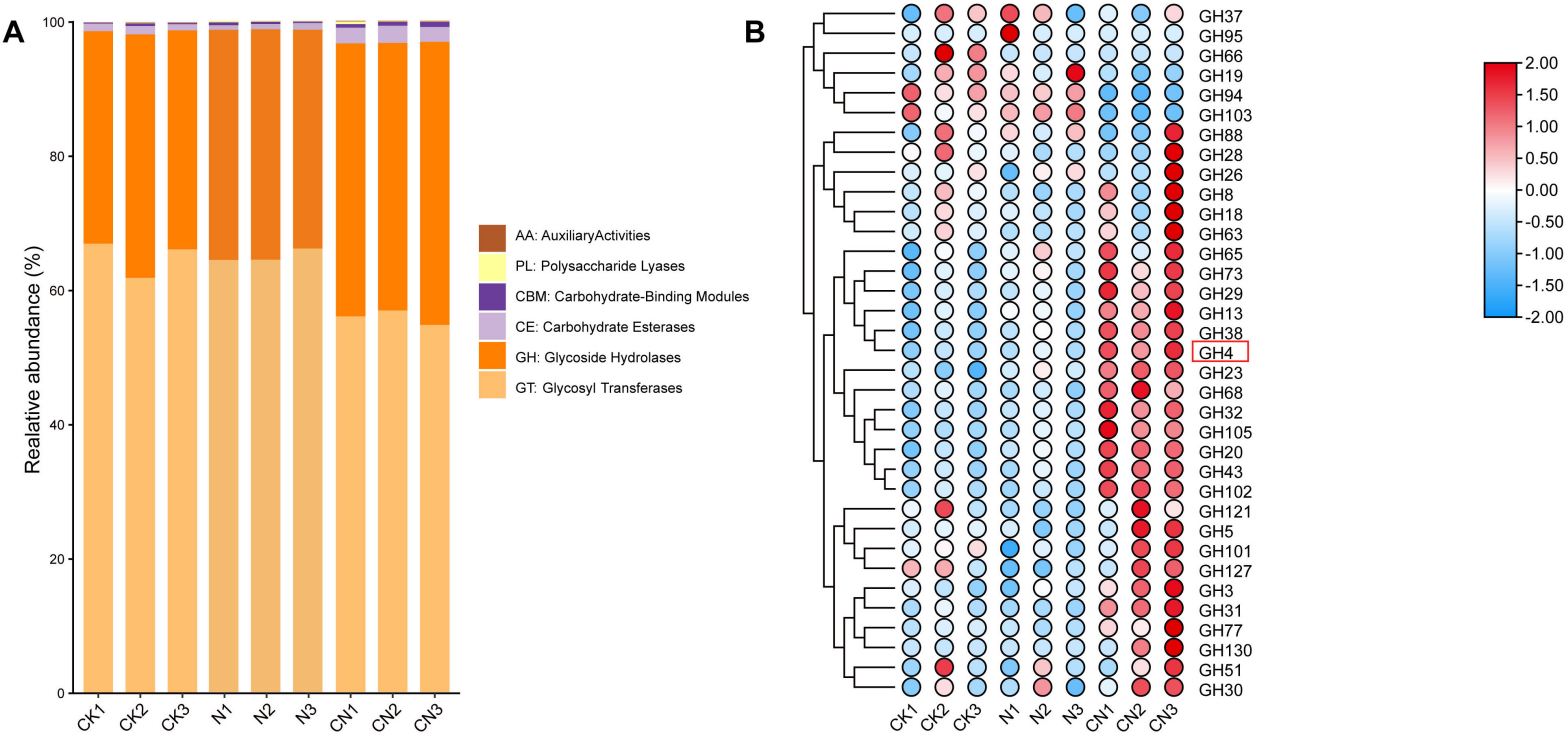
Functionally annotated analysis of the gut flora of *A. mellifera* from three groups. **(A)** CAZy annotation results; **(B)** Heatmap of Glycoside Hydrolases (GH).

Glycoside hydrolases (GH), glycosyl transferases, carbohydrate esterase, carbohydrate-binding modules, polysaccharide lyases, and auxiliary activity families were found in the following order of abundance: CN > N > CK (**Figure 3A**). The abundance of GHs in the CN group was 1.61 times higher than that in the CK group and 1.45 times higher than in the N group. Similarly, the abundance of GH65, GH73, GH29, GH13, GH38, GH4, GH23, GH68, GH32, GH105, GH20, GH43, GH102, GH121, GH5, GH101, GH127, GH3, GH31, GH77, GH130, GH51, and GH30 families in the CN group was elevated (**Figure 3B**).

### Multivariate analysis of identified metabolites in the gut of CK, N, and CN

**Figure 4:**
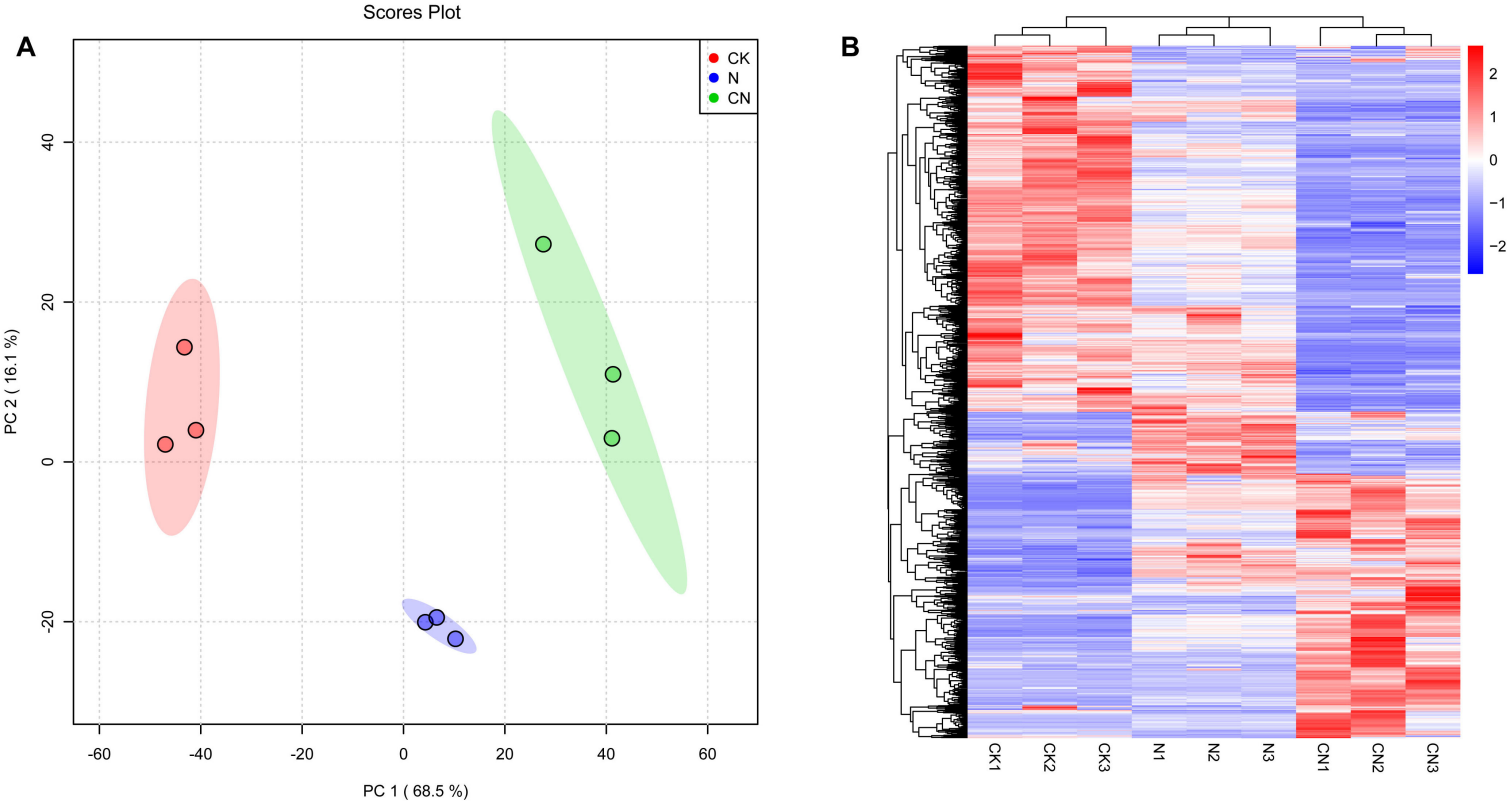
Multivariate statistical analysis of gut metabolites in *A. mellifera* foragers. **(A)** Plot of PCA scores of gut metabolites of *A. mellifera* in CK, N and CN groups; **(B)** Hierarchical cluster analysis heat map of gut metabolites in *A. mellifera* in CK, N and CN groups.

**Figure 4A** presents PCA graph scores for the CK, N, and CN groups. Each point represents a sample, the distance between points represents the similarity between samples, and ellipses are 95% confidence intervals. **Figure 4A** presents that the three groups were independently distributed, with no overlap, indicating intergroup differences among CK, N, and CN groups.

Hierarchical cluster analysis allows for classifying metabolites with the same characteristics into groups. It determines the degree of variation in the content of metabolites with the same characteristics within a group. This analysis revealed differences in gut metabolites of *A. mellifera* foragers in CK, N, and CN groups. Heat maps exhibited that CK, N, and CN groups were categorized into three different profiles, suggesting that the consumption of *C. oleifera* honey and the source of the bee species significantly affected the metabolites in the guts of foragers (**Figure 4B**).

### Mannotrsiose, raffinose, and stachyose levels in the gut of CK, N, and CN

**Figure 5:**
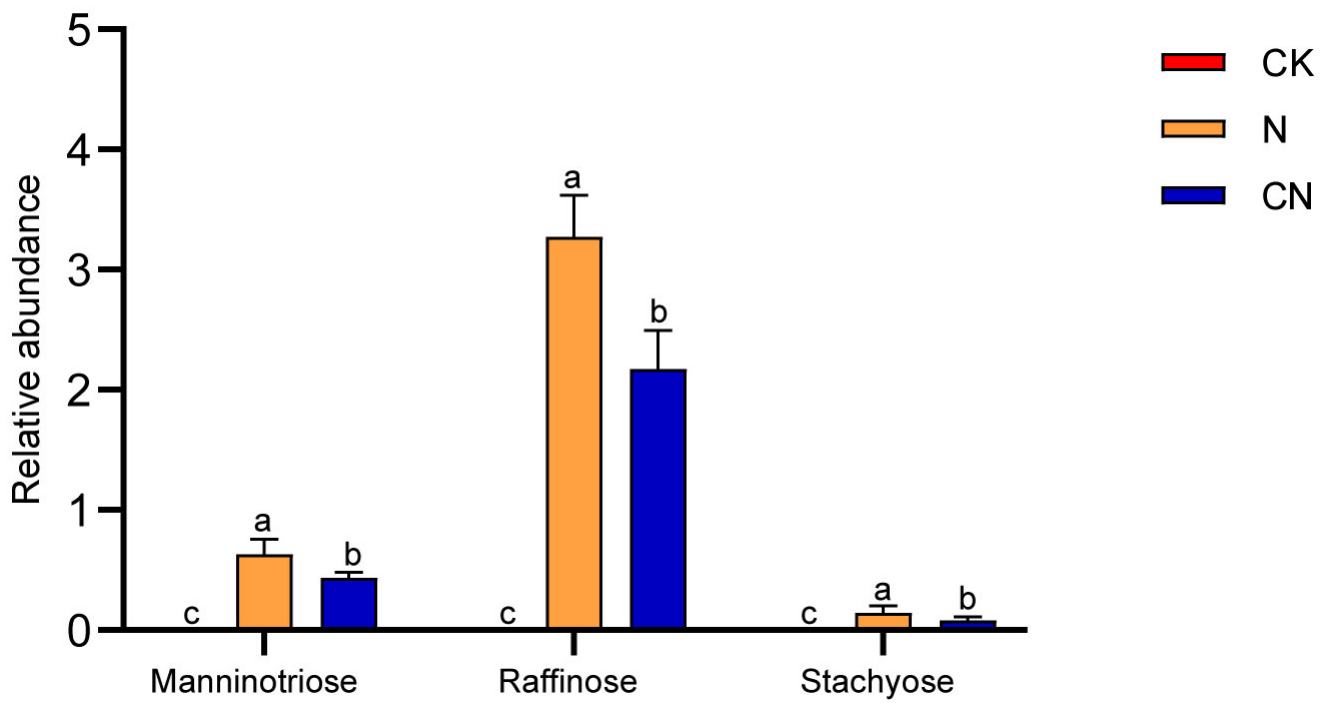
Mannanotriose, raffinose and stachyose relative abundance in gut of *A. mellifera* foragers in CK, N and CN groups. The same lowercase letter in the three columns indicates data no significant difference (*P* > 0.05), and different lowercase letters indicate data significant differences (*P* < 0.05).

**Figure 5** reveals that mannanotriose, raffinose, and stachyose were undetected in the guts of foragers in the CK group. However, mannanotriose, raffinose, and stachyose levels were significantly lower in the guts of foragers in the CN group than in those in the N group (*P* < 0.05). This suggests that the three oligosaccharides accumulated in the guts of the foragers from the CN group.

## Discussion

Honey bee gut flora has multiple functions, such as nutrient metabolism, enhancing immune function, inhibiting pathogenic bacterial growth, and affecting the neurological function and behavior of honey bees (Huang et al., 2023; Liberti and Engel, 2020; Zhang et al., 2022). Previous studies have demonstrated that worker larvae of *A. mellifera* digest manninotriose, raffinose, and stachyose in *C. oleifera* honey, whereas adult worker bees can partially digest these three toxic oligosaccharides (Li et al., 2022). This phenomenon may be primarily due to the low abundance of flora in larvae (Kešnerová et al., 2017). In this study, we found that the abundance of *G. apicola* in the gut of the CN group, which had been present in *C. oleifera* forest for 14 years, was significantly higher than that of the N group. *G. apicola* abundance in the gut of the N group was significantly higher than that of the CK group, which did not consume *C. oleifera* honey 24 h after consuming *C. oleifera* honey. This suggests an upregulation of gut *G. apicola* abundance in response to toxic oligosaccharides in *C. oleifera* honey for the foragers of the N group.

*G. apicola* is an important pectin-degrading bacterium that encodes most of the enzymes required to degrade and use the pectin skeleton but lacks pectin demethylase (Zheng et al., 2019). It breaks down polysaccharides, such as pectin, into small molecules, such as monosaccharides or oligosaccharides, which can be absorbed and used by the bee gut to provide energy and nutrients (Lee et al., 2018). Moreover, when combined with other gut bacteria, such as Bifidobacterium, it can degrade plant polysaccharides more efficiently to meet the nutritional needs of honey bees (Tang et al., 2024). Manninotriose, raffinose, and stachyose are alpha-galactosides (α-Gal), and the *G. apicola* genome includes an α-Gal gene (gene id: 29849712, NZ_CP007445.1) encoding an enzyme that hydrolyzes the α-galactoside bond. This result implied that the upregulated abundance of *G. apicola* in the gut of CN and N group foragers contributes to helping the host break down the three toxic oligosaccharides. *G. apicola* metabolizes α-galactoside by a similar mechanism as it metabolizes mannose (Zheng et al., 2016). Hayakawa et al. (1990) reported that 58 strains of human fecal flora from seven genera including *Bifidobacterium* spp. and *Lactobacillus* spp. were able to utilize α-galactoside. Thus, *Bifidobacterium* spp. and *Lactobacillus* spp. in the gut of the *A. mellifera* may also help the host to metabolize toxic α-galactosides, but their metabolic mechanisms differ from those of *G. apicola* possibly due to the genetic stability of the gut bacteria of the *A. mellifera* (Liberti et al., 2022).

α-Gal (EC 3.2.1.22) are glycoside hydrolases that can specifically hydrolyze the α-Gal bond, such as the cotton-glucose family of oligosaccharides, galactomannans, acacia bean gum, guar gum, and others (Katrolia et al., 2014). Based on the amino acid sequence, the α-Gal CAZy database is attributed to families 4, 27, 36, 57, 97, and 110 of the GH family (Anggraeni et al., 2008). Most α-Gal belong to GH27 and GH36, and these two families of enzymes are the most widely studied (Huang et al., 2018). In contrast, the GH4 α-Gal family is primarily derived from archaea and bacteria. Ascribed to substrate specificity, α-Gal has been categorized into two groups. One class acts against low-molecular-weight substrates, such as 4-nitrophenyl-α-D-galactopyranoside, melibiose, raffinose, and stachyose, whereas the other class acts against highly polymerized galactomannans and low-molecular-weight substrates (Álvarez-Cao et al., 2019). The CAZy annotation results revealed that GH4 abundance was significantly upregulated in the gut flora of bees in the CN group than in the N and CK groups. This result indicated that the gut flora of *Apis mellifera* in *C. oleifera* forest would evolve towards a dominant ecological niche to metabolize toxic oligosaccharides, thereby prolonging the lifespan of their hosts and themselves. After 24 h of *C. oleifera* honey feeding, manninotriose, raffinose, and stachyose accumulation was significantly lower in the gut of foragers in the CN group than in the N group. It was also confirmed that *G. apicola* and α-Gal-secreting flora in the gut of the CN group helped metabolize toxic oligosaccharides.

In the future, upregulating the abundance of *G. apicola* and α-Gal-secreting flora in the gut of *A. mellifera* may ameliorate or resolve the challenge of poisoning in *A. mellifera* following visits to *C. oleifera*. Increasing fruiting rate and camellia oil production may also improve the pollination efficiency of *C. oleifera*.

## Data Availability

The original contributions presented in the study are included in the article/supplementary material. Further inquiries can be directed to the corresponding author.
