# Supplementary material for: Microorganism changes in the gut of Apis mellifera surviving for the long term in Camellia oleifera forests
Source: Front Cell Infect Microbiol. 2025 Jun 12;15:1608835. doi: 10.3389/fcimb.2025.1608835 (PMC12198164; doi:10.3389/fcimb.2025.1608835)
Supplement: Supplementary file 1 [file Table1.docx]

**Metagenome data quality control, assembly and annotation**

*Sequence quality control and genome assembly*

Quality control of the raw data was conducted using fastp (Chen et al. 2018), including the removal of adapter sequences and low-quality reads. The reads were filtered if they had an adapter sequence, an N (uncertain base) ratio > 10%, and a content of low-quality bases (Q ≤ 10) > 50%. The clean reads were mapped to the *A. mellifera* reference genome using Burrows-Wheeler Aligner (Li and Durbin 2009)(version 3.0) to identify and remove host-originated reads.

Then, these high-quality reads were assembled into contigs using MEGAHIT (Li et al. 2015) (parameters: kmer_min = 47, kmer_max = 97, step = 10) (version 1.1.2), with succinct de Bruijn graphs. Contigs > 800 bp in length were selected as the final assembly results.

*Gene prediction, taxonomy, and functional annotation*

Open reading frames (ORFs) in contigs were identified using Prodigal (Hyatt et al. 2010). Predicted ORFs with lengths > 100 bp were retrieved and translated into amino acid sequences using the NCBI translation table.

A non-redundant gene catalog was constructed using CD-HIT (http://www.bioinformatics.org/cd-hit/, version 4.6.1) with 90% sequence identity and 90% coverage. After quality control (Fu et al. 2012), the reads were mapped to a non-redundant gene catalog with 95% identity using Bowtie 2 (Langmead and Salzberg 2012), and the gene abundance in each sample was evaluated (Qin et al. 2010).

*Species and function annotation*

Representative sequences of the non-redundant gene catalog were annotated based on the NCBI Non-redundant database (version 2021.11) using blastp, as implemented in Diamond (version 0.8.35) for taxonomic annotation (Buchfink et al. 2015), with an e-value cutoff of 1×e−5. A cluster of orthologous groups of proteins annotated for representative sequences was performed using Diamond against the Evolutionary genealogy of genes: Non-supervised Orthologous Groups database (version 4.5.1), with an e-value cutoff of 1×e−5. Annotation was conducted using Diamond (version 0.8.35) against the Kyoto Encyclopedia of Genes and Genomes (KEGG) database (version 94.2), with an e-value cutoff of 1×e−5.

CAZy annotation was conducted using hmmscan (http://hmmer.janelia.org/search/hmmscan) against the CAZy database (http://www.cazy.org/), with an e-value cutoff of 1×e−5.

**Chromatography and mass spectrometry analysis methods**

An ACQUITY UPLC T3 column (100 × 2.1 mm, 1.7 µm, Waters, UK) was used for reversed-phase separation. The column oven was maintained at 50 °C. The flow rate was 0.3 mL/min, and the mobile phase consisted of solvents A (0.1% formic acid in water) and B (0.1% formic acid in ACN). Gradient elution conditions were set as follows: 0–0.5 min, 5% B; 0.5–2.5 min, 5% to 70% B; 2.5–7.5 min, 70%–100% B; 7.5–9.0 min, 100%, 9.0–9.5 min, 100%–5%; 9.5–12 min, 5% B.

Sample metabolic analytes flowing from the column were collected in positive and negative modes using high-resolution mass spectrometry triple time of flight 5600+. The detailed parameters are as follows: ion source gas 1, 50 psi; ion source gas 2, 50 psi; curtain gas, 35 psi; source temperature, 500 °C; ion spray voltage floating, 5500 and –4500 V (positive and negative); declustering potential (DP), ± 80 V (positive and negative); TOF MS scan m/z range, 60–1200 Da; product ion scan m/z range, 25–1200 Da; TOF MS scan accumulation time, 0.25 s/spectra; product ion scan accumulation time, 0.03 s/spectra. Secondary mass spectrometry was performed using information-dependent acquisition in the high-sensitivity mode (CE: 30 V ± 15).

**References**

Buchfink, B., Xie, C., and Huson, D. H. (2015). Fast and sensitive protein alignment using DIAMOND. *Nat. Methods* 12, 59-60. doi: 10.1038/nmeth.3176

Chen, S., Zhou, Y., Chen, Y., and Gu, J. (2018). fastp: an ultra-fast all-in-one FASTQ preprocessor. Bioinformatics 34, i884–i890.

Fu, L., Niu, B., Zhu, Z., Wu, S., and Li, W. (2012). CD-HIT: accelerated for clustering the next-generation sequencing data. *Bioinformatics* 28, 3150-3152. doi: 10.1093/bioinformatics/bts565

Hyatt, D., Chen, G. L., LoCascio, P. F., Land, M. L., Larimer, F. W., and Hauser, L. J. (2010). Prodigal: prokaryotic gene recognition and translation initiation site identification. *BMC Bioinformatics* 11, 1-11. doi: 10.1186/1471-2105-11-119

Langmead, B., and Salzberg, S. L. (2012). Fast gapped-read alignment with Bowtie 2. *Nat. Methods* 9, 357-359. doi: 10.1038/nmeth.1923

Li, D., Liu, C. M., Luo, R., Sadakane, K., and Lam, T. W. (2015). MEGAHIT: an ultra-fast single-node solution for large and complex metagenomics assembly via succinct de Bruijn graph. *Bioinformatics* 31, 1674-1676. doi: 10.1093/bioinformatics/btv033

Li, H., and Durbin, R. (2009). Fast and accurate short read alignment with Burrows–Wheeler transform. *Bioinformatics* 25, 1754-1760. doi: 10.1093/bioinformatics/btp324

Qin, J., Li, R., Raes, J., Arumugam, M., Burgdorf, K. S., Manichanh, C., Nielsen, T., Pons, N., Levenez, F., and Yamada, T. (2010). A human gut microbial gene catalogue established by metagenomic sequencing. *Nature* 464, 59-65. doi: 10.1038/nature08821
